# Study on the Compression Effect of Clothing on the Physiological Response of the Athlete

**DOI:** 10.3390/ma15010169

**Published:** 2021-12-27

**Authors:** Marianna Halász, Jelka Geršak, Péter Bakonyi, Gabriella Oroszlány, András Koleszár, Orsolya Nagyné Szabó

**Affiliations:** 1 Institute for Industrial Product Design, Sándor Rejtő Faculty of Light Industry and Environmental Protection Engineering, Óbuda University, Doberdó u. 6, H-1034 Budapest, Hungary; oroszlany.gabriella@uni-obuda.hu (G.O.); koleszar.andras@uni-obuda.hu (A.K.); szabo.orsolya@uni-obuda.hu (O.N.S.); 2 Research and Innovation Centre for Design and Clothing Science, Faculty of Mechanical Engineering, University of Maribor, Smetanova ulica 17, SI-2000 Maribor, Slovenia; jelka.gersak@um.si; 3 Department of Polymer Engineering, Faculty of Mechanical Engineering, Budapest University of Technology and Economics, Műegyetem rkp. 3, H-1111 Budapest, Hungary; bakonyi@pt.bme.hu

**Keywords:** clothing physiology, tight-fitting sportswear, running test on a treadmill, thermal comfort, skin temperature, perspiration

## Abstract

The study aimed to analyze whether the high compression of unique, tight-fitting sportswear influences the clothing physiology comfort of the athlete. Three specific sportswear with different compression were tested on four subjects while they were running on a treadmill with increasing intensity. The compression effect of the sportswear on the body of the test persons, the temperature distribution of the subjects, and the intensity of their perspiration during running were determined. The results indicate that the compression effect exerted by the garments significantly influences the clothing physiology comfort of the athlete; a higher compression load leads to more intense sweating and higher skin temperature.

## 1. Introduction

Companies develop advanced high-tech, often high-compression sportswear for professional athletes [1,2,3,4,5,6]. Researches show that such sportswear can increase the performance of the athlete [7,8,9,10,11,12,13,14,15,16,17,18]. As a healthy lifestyle involves regular exercise, similar special garments are available for amateur and grassroots sports.

Sportswear has undergone enormous improvement since ancient times. In the ancient Olympic Games, men competed without clothing because they believed the best performance could be achieved this way. Until the 18th century, the manufacturing cost of clothes was so high that only the nobility and wealthy people could afford more clothing, especially for sports. During the Industrial Revolution, however, the textile industry started to develop at a tremendous rate, and so not only rich people could afford a garment or garments intended for sport. Until the 19th century, doing sports was mainly a privilege of men, but this did not bring about considerable changes in sportswear. Women started to play sports in higher numbers in the second half of the 19th century. The demand for women’s sportswear started the development of sportswear, continuing intensively to this day [19,20,21,22].

The development of sportswear is directly related to the requirements of the sport, the nature and duration of the activities, and the requirements for adequate thermo-physiology comfort of the athlete [23,24,25,26,27,28,29,30]. In addition, scientists are examining the possibility of increasing performance through the compression that the garment exerts on the athlete’s body [7,8,9,10,11,12,13,14,15,16,17,18]. In recent years, fibers for the manufacture of premium sportswear have been extensively researched and considerably improved. With the advancement of technology and the development of high-performance materials, sportswear now has greatly improved properties. Special emphasis is given to high aerodynamic and absorption properties, air and water permeability, strength, and adhesion. Much has also been performed in the field of design [31]. Sportswear is designed not to restrict the athlete’s activity but provide them physical support while exercising [32].

These sportswear fabrics are usually composed of polyester or polyamide fibers and elastomer fibers. The light and strong polyester or polyamide fibers provide the necessary strength and clothing physiology parameters. The elastomer fibers, capable of large, completely elastic deformation, ensure that the garment completely fits the athlete’s body during exercise and provides compression on it. The knitted fabric structure also facilitates elastic deformation due to the interconnecting loops [1,2,3].

Compression sportswear is a tight-fitting, compressive form of clothing utilizing the material’s elasticity. Professional athletes wear compression suits to improve their athletic performance and speedy recovery from injury. Compression garments are used in high-performance sports such as running, skiing, swimming, and cycling [33]. It has been reported that compression garments improve the perception of muscle damage and increase performance in endurance tests [34]. Compression pants help improve athletic performance and reduce injuries by reducing muscle oscillations [35]. The use of compression stockings minimizes the risk of injury from the overall impact of physical exertion [34].

This case study aims to analyze whether the compression of the tight-fitting sportswear affects the clothing physiology comfort of the athlete and their motion parameters. In our literature search, we did not find any source that investigated the relationship between the compression of sportswear and the clothing physiology of an athlete. Therefore, we believe that the idea that this is worth exploring is novel.

## 2. Materials and Methods

We tested three tight-fitting sportswear with different compression. In the study, four subjects wore these tight-fitting sports garments while running with increasing intensity on a treadmill.

### 2.1. The Test Persons

We included four girls (TP1, TP2, TP3, and TP4) of similar age and body type in the study. The age, body mass, and main body measurements of the test subjects are detailed in Table 1.

The fitness levels of the girls are comparable. Currently, they do sports as a hobby, but as children and teenagers, they were active athletes. They exercise regularly every week, so the running intensity and interval in the tests matched their fitness level.

### 2.2. Materials

For our purpose, three tight-fitting sportswear were used: one ready-made sportswear made of polyester/elastane knitted fabric and two made-to-measure sportswear sets made of polyamide/elastane knitted fabric. Table 2 gives their designation and material composition.

**Figure 1:**
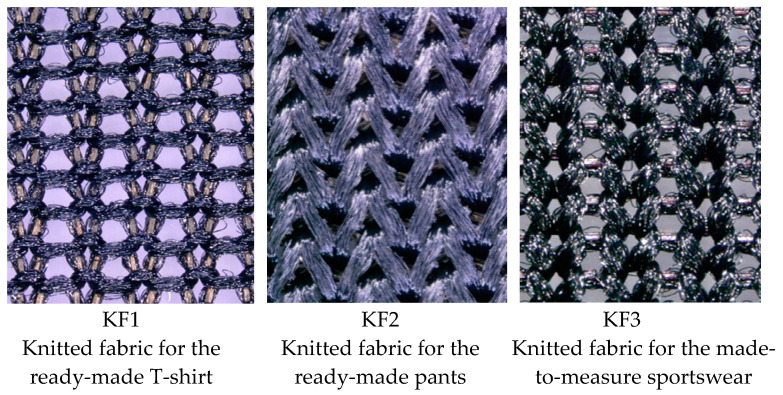
The structure of the knitted fabrics used (microscopic images at 100× magnification).

All three materials are a single weft-knitted fabric and contain a high percentage (20–26%) of elastane fibers. The structure of the knitted fabrics used is shown in Figure 1. The knitted structure with integrated elastane yarn ensures large-scale, multiaxial elastic deformation for the fabrics. In addition, the polyester (PES) and polyamide (PA) fibers provide strength and abrasion resistance and quickly wick sweat away from the body and allow it to evaporate, as they can retain very little moisture.

Although the composition of the studied knitted fabrics is different, they can be used for our research purpose. This is because, although the properties of PA and PES fibers are not the same, the pressure exerted by sportswear made from these knitted fabrics is determined by the elastane fibers, which are present in a high proportion (20–26%) in the knitted fabrics in addition to PA and PES fibers.

The Ready-made Sportswear was bought in sizes S and M, corresponding to the size of the test persons and contains a long-sleeved T-shirt made from knitted fabric KF1 and pants made from knitted fabric KF2. The two made-to-measure sports suits (a long-sleeved T-shirt and pants) were constructed and made by us according to the cut of Ready-made Sportswear from the knitted fabric, KF3. They differ only in ease allowance (Table 2).

The Made-to-measure Sportswear 1 and 2 are made based on the test persons’ body measurements. To ensure comparability, we constructed the patterns of the Made-to-measure Sportswear 1 and 2 to be similar to the bought Ready-made Sportswear. To achieve a compression effect of the sportswear, we have constructed the pattern for the tailored sportswear with negative ease allowance. This negative ease allowance was 1% in the case of the Made-to-measure Sportswear 1 and 5% for the Made-to-measure Sportswear 2, reducing the pattern size by 1% and 5% to under body size, respectively.

Table 3 summarizes the most important properties of the knitted fabrics used. The tests were performed in the Research and Innovation Centre for Design and Clothing Science at Faculty of Mechanical Engineering, University of Maribor, by standard conditions, according to ISO 139 (a temperature of 20 °C and a relative humidity of 65%). The following instruments were used: a Hildebrand thickness gauge, an Akustron Air-Permeability Measuring Instrument, an HC103/01 Moisture Analyzer, a KES-F7 Thermo Labo II for thermal properties, and a Zwick 005 universal testing machine for tensile tests (Zwick Roell GmbH, Ulm, Germany). The static immersion water absorption of the knitted fabrics used was determined according to the ASTM D 583-63 standard.

### 2.3. Test Protocol

The four selected healthy girls participated in the experiment, where we studied the compression effect of clothing on the physiological response of the test subjects. They were selected to be approximately the same age (their average age is 21.0 ± 2.2 years), roughly the same size (body mass 63.3 ± 2.1 kg, height 170.5 ± 2.5 cm), and a similar level of fitness. This way, despite the relatively low number of test persons, we can draw sufficiently well-founded conclusions about the given population.

The wear trial tests were performed under the following ambient conditions: an ambient temperature of 24 °C and relative humidity of 40%.

The test persons wore the previously presented sportswear and ran on a treadmill at increasing intensity. The tests were carried out in the Motion Laboratory of the Department of Mechatronics, Optics and Mechanical Engineering Informatics, and the Biomechanical Research Centre of the Faculty of Mechanical Engineering of the Budapest University of Technology and Economics. The Motion Laboratory also allowed the biomechanical examination of the motion characteristics of the test persons. These results are presented in a separate paper [36].

The test subject performed the same physical activities in each test; a 15-min run, in which running speed was increased every 3 min in the following order:

− 3 min: Load I (running speed of 4 km/h);
− 3 min: Load II (running speed of 7 km/h);
− 3 min: Load III (running speed of 8 km/h);
− 3 min: Load IV (running speed of 10 km/h);
− 3 min: Load V (running speed of 11 km/h).

**Figure 2:**
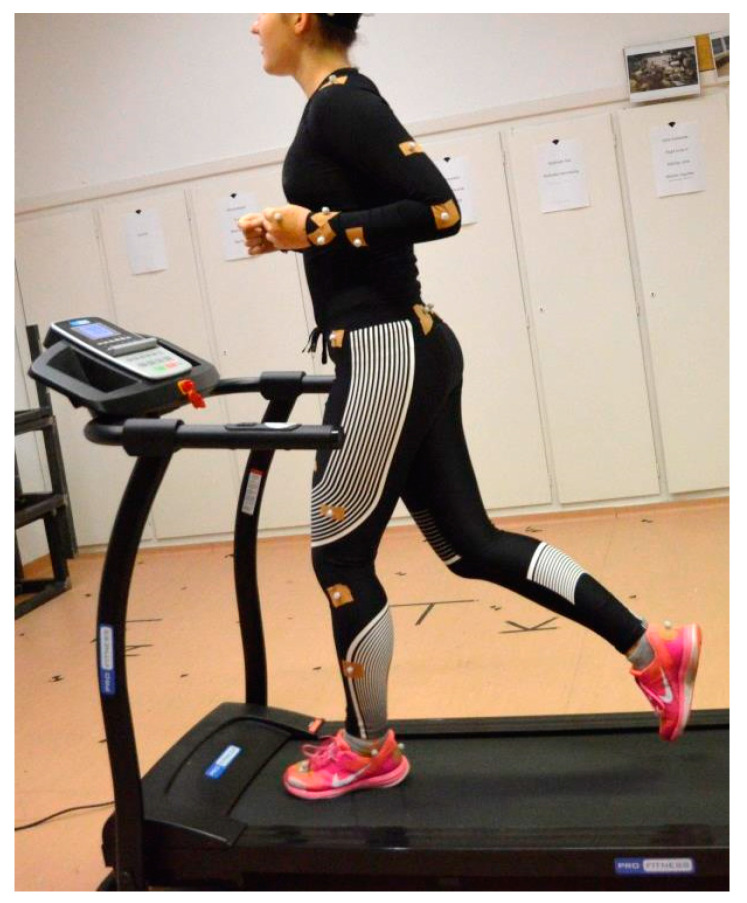
A test person on the treadmill in the Ready-made Sportswear, with the markers for motion tests.

We performed the tests every morning at the same time. The test girls ran on the treadmill wearing another sports suit once each day. Figure 2 shows one of the test subjects in the Ready-made Sportswear while running on the treadmill, with the markers necessary for the movement tests.

According to the study protocol, we determined the compression effect of the suit on the test subjects’ bodies, the temperature distribution of the subjects, and the intensity of their sweating during running. For this purpose, the following measurements were carried out for each test subject while they were wearing their sportswear:

− The compression effect of sportswear on the body (measuring the pressure exerted by the sportswear on the body of the test person);
− Body mass loss due to sweat;
− Thermal imaging during running.

#### 2.3.1. Determination of Pressure Distribution

**Figure 3:**
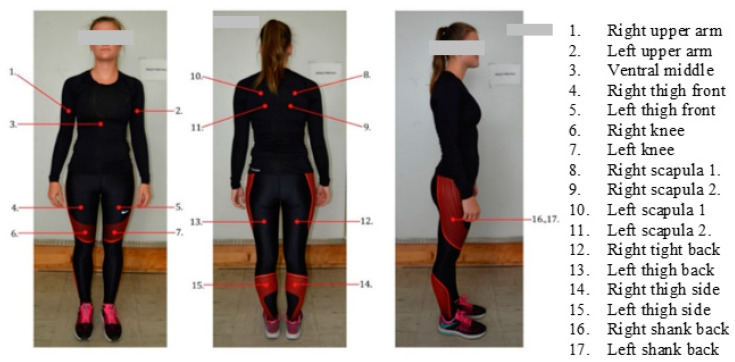
Pressure measurement points.

We measured the compression of sportswear on the test person’s body with the PicoPress tester at 17 points on the body (Figure 3).

Compression was measured on all test persons before the subjects started running. The measurement results are given as mean values of pressure on the upper body (Figure 3, pressure measurement points: 1–3, 8–11) and on the lower body (Figure 3, pressure measurement points: 4–7, 12–17).

#### 2.3.2. Determination of Excreted and Absorbed Sweat

The amount of evaporated sweat was determined based on body mass loss, as the difference in the test subject’s body mass without any clothing, before and after the study. The amount of sweat absorbed in clothing was determined based on the mass of clothing that a test subject was supposed to wear during the test. Test subjects were measured upon completing the study: first dressed, and then the clothing, piece by piece.

The mass of the clothing was measured with an AA Labor MT -XY 6000 (Midwest Microwave Solutions, Hiawatha, IA, USA) balance, which has an accuracy of 0.1 g, while body mass was measured with a Sartorius IS300IGG (Minebea Intec, Hamburg, Germany) balance with an accuracy of 2 g.

#### 2.3.3. Thermal Imaging Analysis

**Figure 4:**
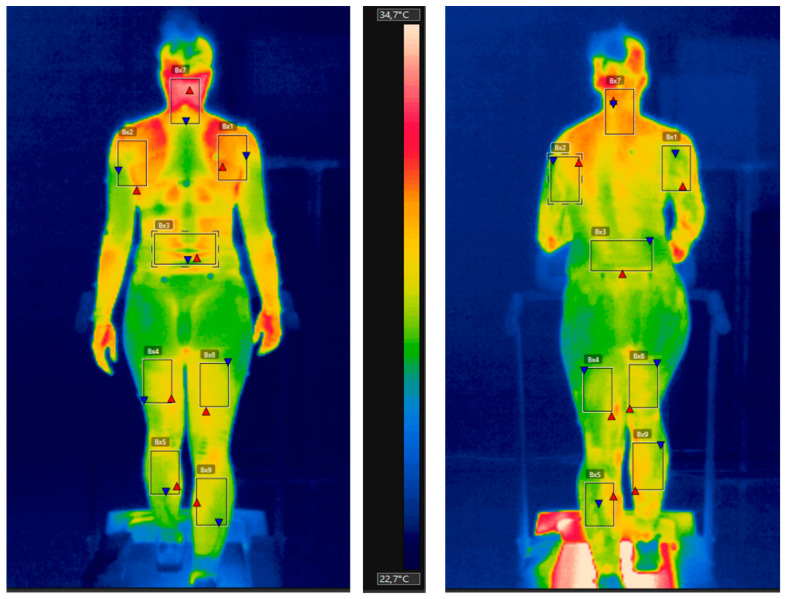
Thermal images at 0.0 min (**left**) and 15.0 min (**right**) with the marked reference areas.

From the clothing physiology point of view, the critical parameter is the thermal load on the subject during running. For this purpose, the body surface temperature was recorded with a FLIR A325sc thermal imaging camera (FLIR Systems, Wilsonville, OR, USA) (Figure 4).

For analysis, we selected eight areas on the body and determined their temperature values based on the thermal images when running speed was changed.

The selected areas were: neck-nape, right and left upper arm, back, right and left thigh, right and left calf. The times of evaluation were: 0 min, 3.0 min, 6.0 min, 9.0 min, 12.0 min, and 15.0 min.

Figure 4 shows the thermal image with the marked selected areas of one test person at 0.0 min and 15.0 min. Except for the neck, all selected areas are covered by sportswear.

## 3. Results and Discussion

Table 4 contains the average pressure values of each sportswear on each test person. (The detailed pressure values for each test person and sportswear at each measurement point are provided in the Appendix A, Table A1). The average pressure values show that the compression of Made-to-measure Sportswear 2 is higher for each test person than in the case of Made-to-measure Sportswear 1 and that the compression of garments constructed with the same amount of reduction in body size is about the same in the case of each test person. In addition, the Ready-made Sportswear has the highest compression, except for Test Person 2, in whose case the compression of the Ready-made Sportswear is between that of the two other, made-to-measure sports suits. It can also be observed that the standard deviation of the measured compression values for the Ready-made Sportswear is greater than that of the two made-to-measure sports suits. This is probably because the Ready-made Sportswear was not made for the size of the test persons; the persons had to choose from the sizes available. For this reason, the compression of the Ready-made Sportswear shows a higher difference between the test persons than that of made-to-measure sportswear.

**Figure 5:**
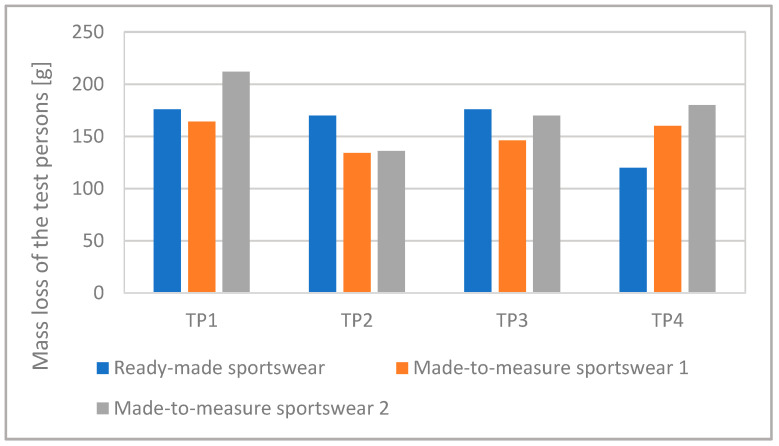
The mass loss of the test persons in the different compression sports suits.

**Figure 6:**
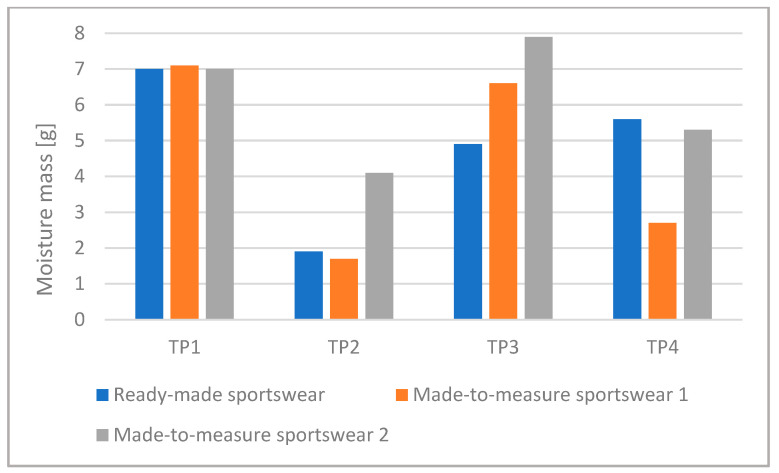
The amount of moisture remaining in the sportswear for each test person and suit.

We analyzed the amount of sweat the test persons produced. Figure 5 shows the difference in body mass without clothing, that is, the mass loss of the test persons. Figure 6 shows the amount of sweat absorbed by the sportswear for each test person and suit.

On average, the test persons lost 100–200 g from their body mass during running (Figure 5). Some of it remained in the sportswear as sweat, and the rest evaporated, cooling the body.

The moisture absorbed by Made-to-measure Sportswear 1 and 2 can be compared well because they were made of the same fabric and the only difference between them is in compression (Figure 6). The higher compression Made-to-measure Sportswear 2 absorbed far more moisture than the lower compression Made-to-measure Sportswear 1 in the case of three test persons (TP2, TP3, and TP4), while in the case of Test Person 1, the two values were nearly identical. The higher compression Made-to-measure Sportswear 2 caused a higher reduction in body mass than the lower compression suit in the case of three test persons (TP1, TP3, and TP4), while with Test Person 2, both sports suits resulted in nearly identical body mass loss (Figure 5). These results indicate that higher compression generated more sweating.

**Figure 7:**
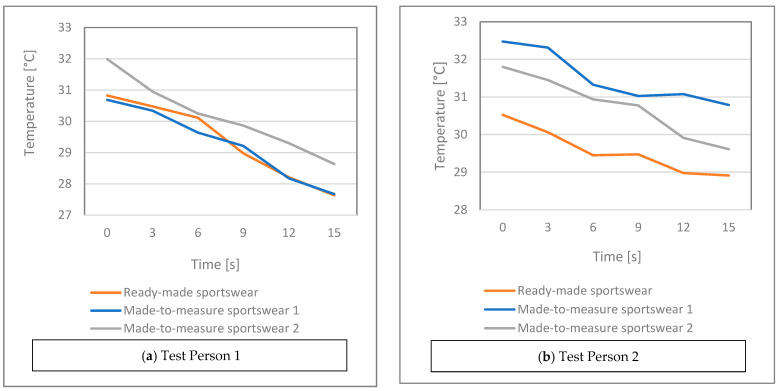

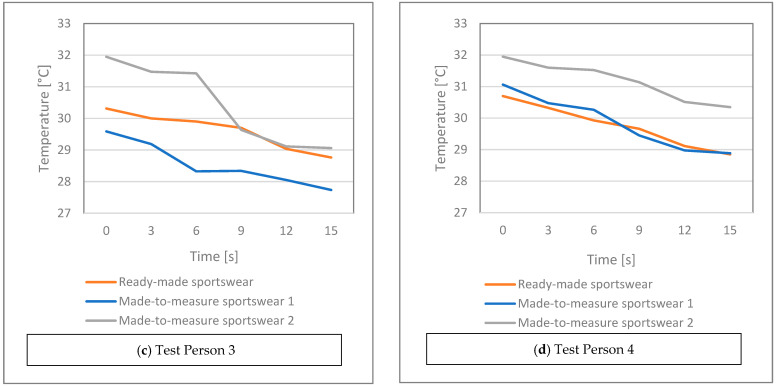
Average body surface temperature as a function of characteristic time points during running for each test person and sports suit.

Figure 7a–d shows the average body surface temperatures as a function of time for each test person and sports suit. As time passes, the average temperature decreases, due to the more intensive sweating, as the evaporating sweat and the moving air caused by running take heat away. The test persons stopped after 15 min.

The results obtained for Made-to-measure Sportswear 1 and 2 can be compared well in the case of thermal imaging as well since they were made from the same fabric; the only difference between them is in compression.

**Figure 8:**
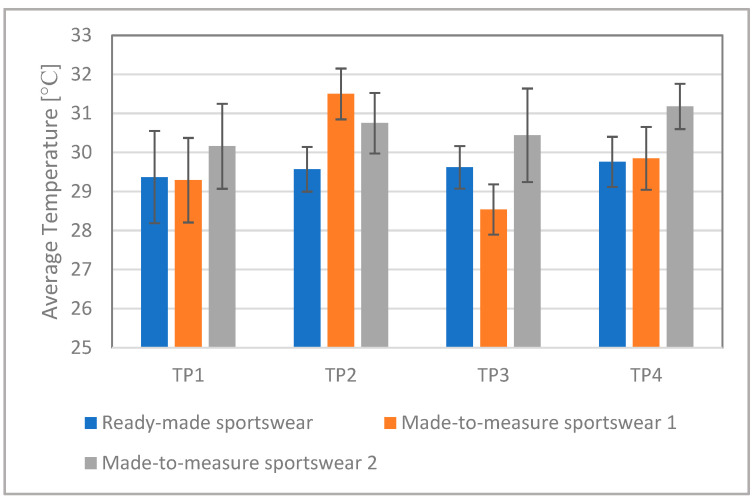
Average body surface temperature calculated for the whole period of running.

For better comparison, Figure 8 shows the average body surface temperature calculated for the whole running period. While wearing the higher compression Made-to-measure Sportswear 2, three test persons (TP1, TP3, and TP4) had a higher body surface temperature during running than in the case of the lower compression Made-to-measure Sportswear 1. In the case of Test Person 2, the opposite was true. These results indicate that higher compression generated higher body surface temperature.

## 4. Conclusions

Our goal was to analyze whether the compression of sportswear on the athlete’s body influences the clothing physiology comfort of the athlete. We included four test persons and used three different types of compression sportswear. We measured the compression of sportswear on the test persons, their body surface temperature, and the intensity of their sweating during running at increasing speed.

Our results indicate that the test persons sweated more in the higher compression Made-to-measure Sportswear 2. Their body surface temperature was higher than in the lower compression Made-to-measure Sportswear 1. We plan to conduct more extensive tests with more test persons to reveal more accurate relationships. Furthermore, we wish to supplement our research with measurements with zero compression sportswear.

However, our initial results strongly indicate that in high-tech sports clothing, the compression applied to increase performance has a considerable effect on the clothing physiology comfort of the athlete. This fact cannot be ignored when sports clothing is designed and fitted.

## Figures and Tables

**Table 1:** The age, body mass, and main body measurements of the test persons.

Designation	Test Persons			
	TP1	TP2	TP3	TP4
Age (years)	23	23	18	24
Body mass (kg)	59.32	66.88	65.25	70.60
Body height (cm)	166	169	171	171
Chest girth (cm)	96	95	92	94
Waist girth (cm)	73	70	71	77
Hip girth (cm)	97	104	99	111
Thigh girth (cm)	55	58	57	64
Lower leg/calf/girth (cm)	35.5	39	38.5	40.5
Inside leg length (cm)	78	76.5	77	79
Dress size upper	S	S	S	M
Dress size pants	S	M	M	M

**Table 2:** The examined sportswear.

Designation	Description	Composition of the Weft-Knitted Fabrics			
		Long-Sleeved T-Shirt		Pants	
Ready-made Sportswear	Ready-made sportswear	KF1	80% Polyester 20% Elastane	KF2	74% Polyester 26% Elastane
Made-to-measure Sportswear 1	Made-to-measure sportswear with 1% body size reduced	KF3	74% Polyamide 26% Elastane	KF3	74% Polyamide 26% Elastane
Made-to-measure Sportswear 2	Made-to-measure sportswear with 5% body size reduced	KF3	74% Polyamide 26% Elastane	KF3	74% Polyamide 26% Elastane

**Table 3:** Properties of the knitted fabrics of the examined sportswear.

Code of Knitted Fabric	Mass (g/m^2^)	Thickness (mm)	Course Density (Piece/10 mm)	Wale Density (Piece/10 mm)	Air Permeability (L/m^2^·s)	Moisture Content (%)	Immersion Water Absorption (%)	Dry Heat Flow (W)	Thermal Resistance R_ct_ (m·^2^K/W)	Force at 5% Elongation * (N/m)
KF1	194.1 ± 1.5	0.397 ± 0.005	30	22	388.6 ± 7.2	3.15 ± 0.27	69.0 ± 2.38	2.07 ± 0.09	0.0719	7.44 ± 0.14
KF2	279.1 ± 5.3	0.497 ± 0.012	25	22	427.2 ± 25.9	1.35 ± 0.02	45.6 ± 0.85	2.01 ± 0.04	0.0636	10.36 ± 0.39
KF3	206.2 ± 0.3	0.356 ± 0.003	28	24	133.0 ± 5.3	2.93 ± 0.15	82.2 ± 1.64	2.13 ± 0.12	0.0721	12.35 ± 0.21

* In course direction.

**Table 4:** Average pressure values for each test person and sports suit.

Examined Sportswear	Body Part	Average Pressure (mmHg)			
		TP 1	TP 2	TP 3	TP 4
Ready-made Sportswear	upper	03.43 ±1.72	2.57 ± 0.79	3.14 ± 1.21	03.72 ± 2.14
	lower	12.08 ±6.13	7.92 ± 4.76	9.75 ± 5.67	10.75 ± 4.86
Made-to-measure Sportswear 1	upper	1.72 ± 1.70	1.86 ± 0.69	2.00 ± 1.00	2.00 ± 1.41
	lower	6.17 ± 3.27	5.83 ± 2.12	6.25 ± 2.60	6.83 ± 2.52
Made-to-measure Sportswear 2	upper	3.43 ± 1.99	3.57 ± 0.98	3.14 ± 1.21	3.43 ± 1.90
	lower	7.25 ± 2.63	8.25 ± 2.99	8.25 ± 2.86	7.67 ± 2.84

## Data Availability

Detailed data can be provided by the corresponding author on request.

## Appendix A

**Table A1:** Detailed pressure values for each test person and sportswear at each measurement point.

**Person**	**TP1**				**TP2**			
**Number of Points**	**Name of Points**	**Pressure (mmHg)**			**Name of Points**	**Pressure (mmHg)**		
		**Ready-made**	**Made-to-Measure 1**	**Made-to-Measure 2**		**Ready-made**	**Made-to-Measure 1**	**Made-to-Measure 2**
1.	Right upper arm	3	2	2	Right upper arm	2	2	3
2.	Left upper arm	4	2	3	Left upper arm	2	2	4
3.	Ventral middle	1	0	1	Ventral middle	2	1	2
4.	Right thigh front	7	5	7	Right thigh front	4	5	7
5.	Left thigh front	6	5	7	Left thigh front	4	4	6
6.	Right knee	7	5	7	Right knee	5	6	6
7.	Left knee	8	6	5	Left knee	5	5	7
8.	Right Scapula 1	2	1	3	Right Scapula 1	3	2	3
9.	Right Scapula 2	5	2	5	Right Scapula 2	3	3	4
10.	Left Scapula 1	3	0	3	Left Scapula 1	2	1	4
11.	Left Scapula 2	6	5	7	Left Scapula 2	4	2	5
12.	Right tight back	14	11	6	Right tight back	5	5	7
13.	Left thigh back	16	11	6	Left thigh back	6	4	8
14.	Right thigh side	6	5	4	Right thigh side	5	4	5
15.	Left thigh side	5	5	4	Left thigh side	4	3	5
16.	Right shank back	18	5	9	Right shank back	16	9	13
17.	Left shank back	17	4	9	Left shank back	14	8	13
18.	Right waist	20	13	10	Right waist	14	8	12
19.	Left waist	21	11	13	Left waist	13	9	10
Average [mmHg]:		8.89	5.16	5.84		5.95	4.37	6.53
Stand. dev. [mmHg]:		6.51	3.85	3.02		4.59	2.61	3.34
**Person**	**TP3**				**TP4**			
**Number of Points**	**Name of Points**	**Pressure (mmHg)**			**Name of Points**	**Pressure (mmHg)**		
		**Ready-made**	**Made-to-Measure 1**	**Made-to-Measure 2**		**Ready-made**	**Made-to-Measure 1**	**Made-to-Measure 2**
1.	Right upper arm	4	2	2	Right upper arm	3	1	3
2.	Left upper arm	4	2	4	Left upper arm	3	2	7
3.	Ventral middle	2	2	2	Ventral middle	1	1	1
4.	Right thigh front	5	5	6	Right thigh front	7	5	5
5.	Left thigh front	6	6	6	Left thigh front	7	5	5
6.	Right knee	7	5	8	Right knee	11	5	6
7.	Left knee	7	5	9	Left knee	8	5	6
8.	Right Scapula 1	3	2	2	Right Scapula 1	6	2	2
9.	Right Scapula 2	2	1	5	Right Scapula 2	4	1	4
10.	Left Scapula 1	2	1	3	Left Scapula 1	2	2	3
11.	Left Scapula 2	5	4	4	Left Scapula 2	7	5	4
12.	Right tight back	7	7	6	Right tight back	7	6	6
13.	Left thigh back	10	9	7	Left thigh back	8	9	5
14.	Right thigh side	4	2	5	Right thigh side	7	4	4
15.	Left thigh side	3	2	5	Left thigh side	7	4	3
16.	Right shank back	18	10	11	Right shank back	14	10	10
17.	Left shank back	17	8	12	Left shank back	14	10	13
18.	Right waist	15	7	13	Right waist	19	10	8
19.	Left waist	18	9	11	Left waist	20	9	9
Average [mmHg]:		7.32	4.68	6.37		8.16	5.05	5.47
Stand. dev. [mmHg]:		5.56	2.98	3.45		5.30	3.21	2.95
